# Medication Discrepancies and Regimen Complexity in Decompensated Cirrhosis: Implications for Medication Safety

**DOI:** 10.3390/ph14121207

**Published:** 2021-11-23

**Authors:** Kelly L. Hayward, Patricia C. Valery, Preya J. Patel, Catherine Li, Leigh U. Horsfall, Penny L. Wright, Caroline J. Tallis, Katherine A. Stuart, Michael David, Katharine M. Irvine, Neil Cottrell, Jennifer H. Martin, Elizabeth E. Powell

**Affiliations:** 1 Centre for Liver Disease Research, Faculty of Medicine, Translational Research Institute, The University of Queensland, 37 Kent Street, Woolloongabba, Brisbane, QLD 4102, Australia; patricia.valery@qimrberghofer.edu.au (P.C.V.); preya.patel1@nhs.net (P.J.P.); leigh.horsfall@health.qld.gov.au (L.U.H.); katharine.irvine@uq.edu.au (K.M.I.); e.powell@uq.edu.au (E.E.P.); 2 Pharmacy Department, Princess Alexandra Hospital, 199 Ipswich Road, Woolloongabba, Brisbane, QLD 4102, Australia; catherine.li@health.qld.gov.au; 3 QIMR Berghofer Medical Research Institute, 300 Herston Road, Herston, Brisbane, QLD 4006, Australia; 4 Department of Gastroenterology and Hepatology, Princess Alexandra Hospital, 199 Ipswich Road, Woolloongabba, Brisbane, QLD 4102, Australia; penny.wright@health.qld.gov.au (P.L.W.); caroline.tallis@health.qld.gov.au (C.J.T.); katherine.stuart@health.qld.gov.au (K.A.S.); 5 School of Medicine and Public Health, University of Newcastle, University Drive, Callaghan, Newcastle, NSW 2308, Australia; michael.david@newcastle.edu.au (M.D.); jenniferh.martin@newcastle.edu.au (J.H.M.); 6 Mater Research, Translational Research Institute, The University of Queensland, 37 Kent Street, Woolloongabba, Brisbane, QLD 4102, Australia; 7 Faculty of Health and Behavioral Sciences, The University of Queensland, St Lucia, Brisbane, QLD 4072, Australia; n.cottrell@uq.edu.au

**Keywords:** clinical pharmacist, medication complexity, medication reconciliation, medication related problems, medication safety

## Abstract

Discrepancies between the medicines consumed by patients and those documented in the medical record can affect medication safety. We aimed to characterize medication discrepancies and medication regimen complexity over time in a cohort of outpatients with decompensated cirrhosis, and evaluate the impact of pharmacist-led intervention on discrepancies and patient outcomes. In a randomized-controlled trial (*n* = 57 intervention and *n* = 57 usual care participants), medication reconciliation and patient-oriented education delivered over a six-month period was associated with a 45% reduction in the incidence rate of ‘high’ risk discrepancies (IRR = 0.55, 95%CI = 0.31–0.96) compared to usual care. For each additional ‘high’ risk discrepancy at baseline, the odds of having ≥ 1 unplanned medication-related admission during a 12-month follow-up period increased by 25% (adj-OR = 1.25, 95%CI = 0.97–1.63) independently of the Child–Pugh score and a history of variceal bleeding. Among participants with complete follow-up, intervention patients were 3-fold less likely to have an unplanned medication-related admission (adj-OR = 0.27, 95%CI = 0.07–0.97) compared to usual care. There was no association between medication discrepancies and mortality. Medication regimen complexity, frequent changes to the regimen and hepatic encephalopathy were associated with discrepancies. Medication reconciliation may improve medication safety by facilitating communication between patients and clinicians about ‘current’ therapies and identifying potentially inappropriate medicines that may lead to harm.

## 1. Introduction

The medication regimen consumed by people with decompensated cirrhosis is often characterized by a variety of therapeutic classes, time-specific and medication-specific dosing instructions, and self-monitoring requirements. Medications are important to manage complications of cirrhosis and portal hypertension, the underlying chronic liver disease (CLD), and comorbid conditions. However, patients are vulnerable when there is miscommunication with health providers about current therapies. Low health literacy, intentional or unintentional non-adherence, and patient–provider miscommunication can result in discrepancies between the medications consumed by patients and the documented list in the medical record [1,2,3]. Discrepancies have medication safety implications; they can complicate care and may lead to harm if a clinician alters treatment—as is frequently required due to the natural history and labile complications of advanced liver disease—without knowledge of the entire medication regimen.

Medication discrepancies affect over 70% of patients in ambulatory care settings [1,2,4,5,6] and are an important source of medication-related harm, including adverse drug events and hospital readmissions [7,8,9]. Discrepant medications can contribute to medication-related problems (MRPs), including drug interactions with new therapies, duplicate drug therapy, or prescription of unnecessary medications to treat side effects of unknown therapies [6,7,8,9,10]. People with decompensated cirrhosis may be especially vulnerable to sequelae of discrepancies due to chronic frailty. Frequent hospital admissions (during which the medication regimen may be changed), contact with multiple community and hospital-based health providers, and patient-driven self-management (including the use of supplements and natural therapies) can further increase the opportunity for discrepancies to arise. Medication reconciliation is one strategy endorsed by the World Health Organization to improve medication safety [11]. However, the effect of medication discrepancies and medication regimen complexity on patient outcomes in people with decompensated cirrhosis has not been explored.

We aimed to (i) characterize medication discrepancies in a cohort of ambulatory patients with a history of decompensated cirrhosis; and (ii) explore medication regimen complexity and frequency of medication regimen changes over time. In order to determine whether reduction in medication discrepancies would be associated with improved outcomes, we also aimed to (iii) evaluate the effectiveness of pharmacist-led medication reconciliation to reduce medication discrepancies; and (iv) explore the relationship between discrepancies and medication-related hospitalization and mortality.

## 2. Results

**Figure 1:**
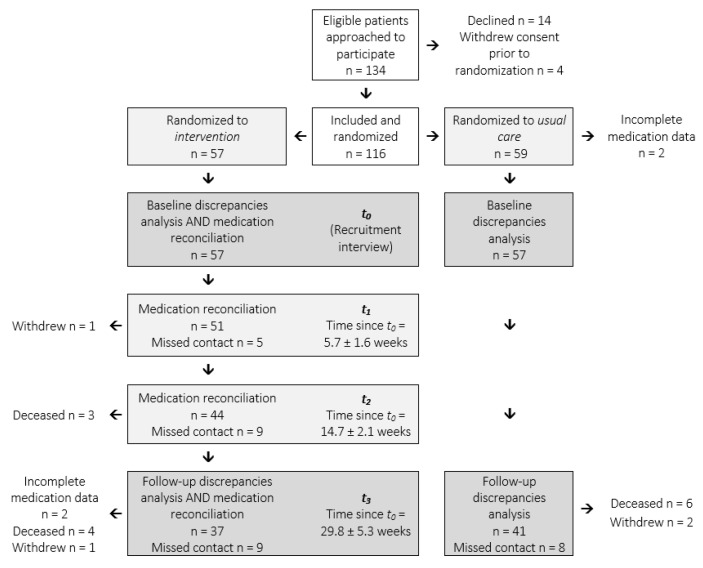
Flow diagram of patient recruitment and follow up timelines.

One-hundred and sixteen patients were recruited when they attended for a routine hepatology review between January 2016 and October 2016. Fifty-nine patients were randomized to receive usual care and fifty-seven to receive the intervention (Figure 1) as previously reported [12]. However, two patients in the usual care group did not disclose their medications at recruitment and were excluded from the current study due to missing data (Table 1).

At recruitment, participants who reported self-managing their medications were taking a smaller median number of medications (7.0 [interquartile range (IQR) 6.0–10.0] vs. 11.0 [IQR 9.0–13.0], *p* < 0.001). A greater proportion of patients with hepatic encephalopathy (HE) reported receiving assistance with medications (i.e., a pharmacy packing aide or assistance from a family member/caregiver) than those without HE (57.5% vs. 17.6%, *p* < 0.001). A greater proportion of patients who self-reported lower levels of education (did not complete high school or any post-school qualification) also reported receiving assistance (45.5% vs. 20.0%, *p* = 0.005).

### 2.1. Prevalence of Medication Discrepancies

At recruitment, patients stated they took a total of 800 medications and clinicians documented a total of 620 medications in the electronic medical record (EMR). Comparison of patient-reported and clinician-documented medications identified a total of 919 distinct medications, of which 730 (79.4%) were associated with a discrepancy; an overall rate of 6.4 discrepancies per patient. For each additional medication taken, the incidence rate of ‘high’ risk discrepancies increased by 10%, reflected by an incidence rate ratio (IRR) of 1.10 (95%CI = 1.04–1.17, *p* = 0.001) (Table 2).

**Figure 2:**
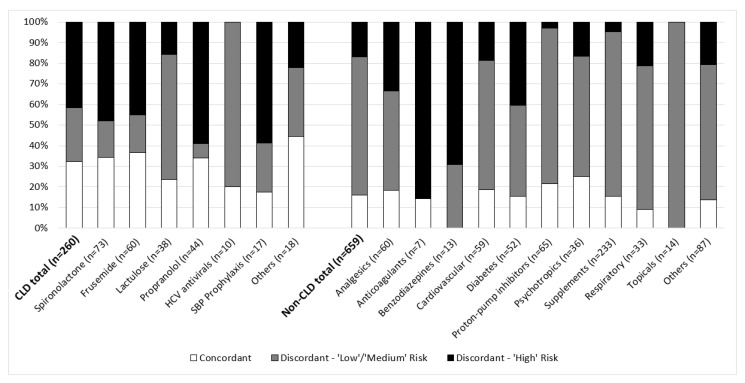
Proportion of discordant CLD and non-CLD medications following comparison of patient-reported and clinician-documented medications at baseline. A total of 919 distinct medications are represented (of which 800 were disclosed by patients and 620 were documented in the EMR).

Approximately half of all discrepancies at recruitment (*n* = 417; 57.1%) were at the name level. A total of 298 medications were stated by the patient but not documented in the EMR, and 119 medications were documented in the EMR by a treating clinician but not disclosed by the patient. Three-hundred and thirteen medications were discordant at the dose/frequency level due to missing documentation or differences in medication dose (*n* = 42; 13.4%), frequency (*n* = 81; 25.9%), or both (*n* = 190; 60.7%). A greater proportion of non-CLD medications were discordant compared to CLD medications (84.1% vs. 67.7%, *p* < 0.001) (Figure 2).

The clinician panel classified 219 discrepancies (30.0%) to be ‘high’ risk for potential harm. Eighty-nine patients (78.1%) had at least one ‘high’ risk discrepancy at baseline. ‘High’ risk discrepancies at the name level (*n* = 146) were most commonly associated with diuretics (*n* = 38; 26.0%), analgesics (*n* = 15; 10.3%) and diabetes medicines (*n* = 15; 10.3%). ‘High’ risk discrepancies at the dose/frequency level (*n* = 73) were most commonly associated with diuretics (*n* = 24; 32.9%) and propranolol (*n* = 13; 17.8%). Overall, the majority of ‘low’ and ‘medium’ risk discrepancies (*n* = 511) were associated with vitamins and supplements (*n* = 186; 36.4%).

### 2.2. Impact of Pharmacist Intervention on Medication Discrepancies

Over the course of the study, intervention patients had a mean of 3.35 ± 0.94 contacts (minimum 1; maximum 4) during which medication reconciliation occurred. At follow-up, the intervention group (*n* = 37) had a significantly lower proportion of discrepant medications and fewer ‘high’ risk discrepancies compared to usual care patients (*n* = 41) (Table 3). There was also lower overall discordance among medication subgroups with known medication safety issues including analgesics (60.7% vs. 100.0%, *p* < 0.001) and narcotics (61.5% vs. 100.0% *p* = 0.013), and fewer discrepancies among vitamins/supplements in the intervention group (67.9% vs. 88.0%, *p* = 0.003).

The final generalized estimating equations model included randomization, time (pre/post-intervention), randomization*time, HE, and total number of medications as an offset variable. From baseline to follow-up, the intervention group had a 45% reduction in the incidence rate of total ‘high’ risk discrepancies (IRR = 0.55, 95%CI = 0.31–0.96, *p* = 0.036) and a 51% reduction in name level ‘high’ risk discrepancies (IRR = 0.49, 95%CI = 0.27–0.89, *p* = 0.018) compared to usual care. Usual care patients had a non-significant increase in the incidence rate of name level ‘high’ risk discrepancies from baseline to follow-up (IRR = 1.56, 95%CI = 0.91–2.69, *p* = 0.106).

### 2.3. Medication Regimen Complexity

Over the four study timepoints, 1650 individual medication entries were identified among intervention patients. A total of 443 changes were made between timepoints: average 3.08 ± 3.21 (t_0_ to t_1_), 3.16 ± 2.82 (t_1_ to t_2_), and 3.77 ± 2.86 (t_2_ to t_3_). Among 34 patients who had four contacts, those taking more medications at t_0_ had more changes to their regimen over the study period (Spearman’s rho = 0.364, *p* = 0.034).

Medications that were associated with the greatest frequency of change included lactulose, insulin, prednisolone, benzodiazepines and antiemetics (Supplementary Table S1). Those with the lowest frequency of change included propranolol, proton pump inhibitors, cardiovascular and antipsychotic/antidepressant medications. The Child–Pugh score was positively correlated with the number of changes made to CLD medications over the study period (Spearman’s rho = 0.339, *p* = 0.050).

The Medication Regimen Complexity Index (MRCI) score was applied to the medication regimen consumed by intervention patients at each time point (Table 4). The mean MRCI score at baseline was 25.59 ± 13.49 and did not substantially change between timepoints. Non-CLD medications contributed a greater proportion of complexity to section B and C scores. Total MRCI score was correlated with number of medications (Pearson’s r = 0.952, *p* < 0.001), comorbidities (Spearman’s rho = 0.644, *p* < 0.001) and medication discrepancies (Spearman’s rho = 0.540, *p* < 0.001), but was not associated with age, gender, CLD etiology or severity.

Among 1650 individual medication entries over the course of the study, CLD medicines had a higher average composite section B and C score than non-CLD medications (2.65 ± 0.94 vs. 2.22 ± 0.93, *p* < 0.001), indicating that the frequency and administration directions associated with CLD medicines are comparatively more complex. Lactulose, propranolol and insulin had significantly more complex frequency directions compared to other medications, due to the need to take these medications several times a day (Supplementary Table S2). Prednisolone, thyroxine and oral hypoglycemics were among the medications that had the most complex additional instruction directions, driven by the need to vary the dose, break the tablets or take more than one unit at a time, and take with respect to food.

### 2.4. Medication-Related Outcomes

During the 12-month follow-up period, 26.3% of patients (*n* = 14 intervention and *n* = 16 usual care) had at least one unplanned medication-related admission and seventeen patients died (14.9%). No death was medication related. There was no association between medication discrepancies and mortality.

Due to the large count of zero admissions, a logistic regression model was used to calculate the odds of having ≥ 1 admission compared to zero. For each additional ‘high’ risk medication discrepancy at baseline, the odds of having ≥ 1 unplanned medication-related admission increased by 25% in a multivariable logistic regression model adjusted for the Child–Pugh score, history of variceal bleeding, and randomization to the intervention arm given the impact of the study intervention on discrepancies over time (Table 5).

In a sub-analysis of participants with complete follow-up medication data (*n* = 37 intervention and *n* = 41 usual care), intervention patients were less likely to have ≥1 unplanned medication-related admission (adj-OR = 0.27, 95%CI = 0.07–0.97, *p* = 0.046) following adjustment for Child–Pugh score (adj-OR = 1.61, 95%CI = 1.12–2.31, *p* = 0.009) and history of variceal bleeding (adj-OR = 5.70, 95%CI = 1.14–28.53, *p* = 0.034).

Within the intervention group, the odds of having ≥ 1 unplanned medication-related admission increased by 6% for every one-unit increase in the t_0_ MRCI score (adj-OR = 1.06, 95%CI = 1.01–1.12, *p* = 0.033), independently of Child–Pugh score (adj-OR = 1.80, 95%CI = 1.12–2.90, *p* = 0.015) and history of variceal bleeding (adj-OR = 6.85, 95%CI = 0.86–54.46, *p* = 0.069). The risk of all-cause mortality (*n* = 8 deaths) increased by 7% (OR = 1.07, 95%CI 1.01–1.13, *p* = 0.028) for every one-unit increase in the t_0_ MRCI score. Addition of baseline MELD score, Child–Pugh score, history of variceal bleeding and hepatocellular carcinoma to the model (as individual variables and in combination) did not result in loss of significance.

## 3. Discussion

There is a high prevalence of medication discrepancies among ambulatory patients with decompensated cirrhosis, likely influenced by the complex and frequently-changing nature of the medication regimen. We have shown that discrepancies are an important medication safety consideration because for each additional ‘high’ risk discrepancy, the odds of having an unplanned medication-related admission within 12-months increased by 25%. Pharmacist-led intervention including medication reconciliation was associated with a reduction in ‘high’ risk discrepancies, which coincided with over 3-fold reduced odds of having an unplanned medication-related admission among intervention patients. Conversely, usual care patients experienced an increase in discrepancies, which suggests that medication reconciliation not only resolves discrepancies but may be protective against accumulation of discrepancies over time.

**Figure 3:**
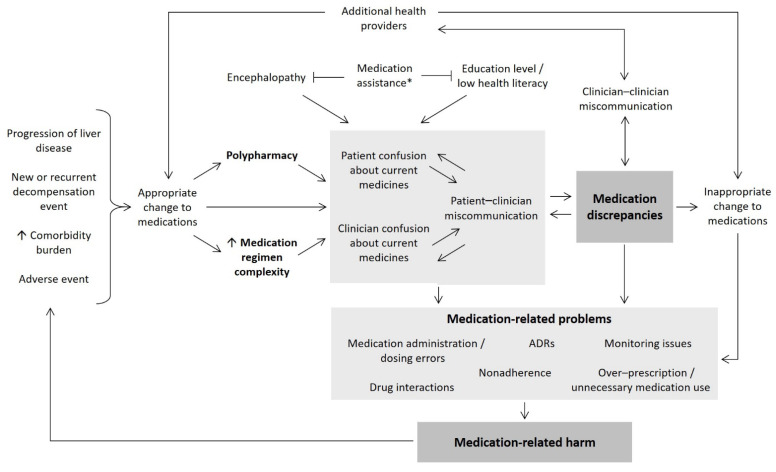
The relationship between medication discrepancies and medication-related harm in people with decompensated cirrhosis. * Medication assistance including (but not limited to) a pharmacy packing aide or assistance from a family member/caregiver. Abbreviation: ADRs, adverse drug reactions.

The mechanism by which discrepancies occur in decompensated cirrhosis is multifactorial (Figure 3). We found discrepancies were associated with taking more medications, which is consistent with studies in other groups [1,4,5,15]. For each additional medication taken in our study, the incidence rate of ‘high’ risk discrepancies increased by 10%. This finding has important clinical implications, as people with decompensated cirrhosis consume on average nine prescription medications each day [16], in addition to over-the-counter and complementary therapies which we have previously reported to have a high (85.5%) discrepancy rate [3]. The number of medications was also positively correlated with MRCI score and the number of regimen changes experienced by intervention patients. Polypharmacy and medication regimen complexity can lead to confusion and increase opportunities for miscommunication about medicines, which can contribute to MRPs and harm [17,18]. While the incidence of adverse drug events was not directly measured in this study, we have previously shown that a higher incidence rate of MRPs is associated with unplanned admissions in decompensated cirrhosis [12]. Interestingly, we found self-management of medications was associated with a lower incidence rate of discrepancies. While this seems counterintuitive, our participants who reported receiving assistance were taking a greater number of medications, were more likely to have HE, and had lower levels of education. This finding highlights the importance of engaging caregivers in chronic disease management.

The high discrepancy rate among non-CLD medicines in our study likely reflects the types of medications discussed between hepatologists and patients during clinical review in the standard model of care. Non-CLD medications represented over three-quarters of the total frequency (MRCI section B) and additional instruction complexity (MRCI section C), which was in line with the proportion of non-CLD medicines consumed by patients and the proportion of changes made. In people with depression, human immunodeficiency virus, diabetes, hypertension, heart failure and transplant populations, approximately two-thirds of the total MRCI score has similarly been attributed to non-disease-specific prescriptions [19,20,21,22]. This finding reinforces that people with chronic diseases often take a range of medications in addition to those prescribed by their specialists, and is particularly relevant given the rising prevalence of metabolic-associated liver disease worldwide. At recruitment, discordance at the name level was highest for topical medicines, respiratory medicines and benzodiazepines, which were predominately prescribed by other health providers. Medication reconciliation identified several intervention patients taking potentially inappropriate medicines such as opioids, benzodiazepines, proton pump inhibitors, and nonsteroidal anti-inflammatory drugs. Use of these medicines among people with cirrhosis is not uncommon [23], which is concerning because these are medicines that may lead to harm due to pharmacokinetic and pharmacodynamic alterations in advanced disease [24]. While not directly measured in this study, deprescribing interventions targeting potentially inappropriate or unnecessary therapies may help to reduce medication regimen complexity and opportunity for discrepancies to arise [25].

Limitations of this study include the relatively small sample size and heterogeneity among the recruited patients. Random allocation was employed to minimize the potential impact of heterogeneity on study findings. Although pharmacist interviews may have permitted disclosure of a greater number of medications and hence discrepancies by intervention patients, we attempted to mitigate this effect by offsetting discrepancy count by the number of medications in the final generalized estimating equations model. Strengths of the study include the prospective design and use of a multidisciplinary panel of clinicians that systematically reviewed and categorized discrepancy risk.

## 4. Materials and Methods

A multifaceted pharmacist-driven intervention targeted to adults with decompensated cirrhosis was implemented as a randomized-controlled trial in a hepatology outpatient center [12]. Eligible patients were invited to participate when they attended for routine follow-up at one of seven concurrent general hepatology clinics. Patients were randomized (1:1 ratio) by allocation concealment to receive the intervention or usual care. Usual care participants received routine review and education by their hepatologist according to the standard model of care in the hepatology clinic throughout the study period. Intervention participants received up to four additional contacts (at t_0_, t_1_, t_2_ and t_3_) over a six-month period from a clinical pharmacist (K.H.) in person or via telehealth, in addition to usual care. During these contacts, patients received medication reconciliation and education tailored to individual needs. Informed written consent was obtained from all participants. 

### 4.1. Data Collection

Participants completed a survey questionnaire at baseline (t_0_) and follow-up (t_3_; approximately six months post-recruitment). The questionnaire asked patients to list their current medications, including dose, frequency and indication for therapy. Patients’ caregivers and/or family members were involved if they assisted with medication management to reflect a “real-world” clinical scenario. For intervention patients, the pharmacist used a structured data collection tool to document medications disclosed verbally during interviews (at t_0_, t_1_, t_2_, and t_3_) and additional medications identified during reconciliation by third sources. Clinician documentation of ‘current’ medications, clinical and demographic information was obtained from patients’ medical records. Outcome data were collected at 52 weeks from medical records and the Queensland Hospital Admitted Patient Data Collection (QHAPDC) registry, which contains information on all hospital episodes of care for patients admitted to all Queensland public and private hospitals.

### 4.2. Measures

#### 4.2.1. Medication Discrepancies

Medication discrepancies were examined at name level, dose/frequency level, and overall. Medications were considered discrepant at the name level if the medication was identified by the patient or clinician, but not both. Medications were considered discrepant at the dose/frequency level if the medication was named, but the patient and clinician listed a different dose and/or frequency of administration. Missing documentation was considered a discrepancy. Documentation by the clinician of intentional changes to current therapies made during review was not considered a discrepancy.

Medications were classified as therapies for CLD, including diuretics, lactulose, rifaximin, propranolol, antiviral therapies for hepatitis B and C, and spontaneous bacterial peritonitis prophylaxis; or non-CLD medications. The clinical significance of discrepancies was independently examined by a panel of clinicians (including a hepatologist, hepatology fellow, pharmacologist/general physician and two pharmacists) using a risk matrix (see Appendix A), which categorized overall risk to be ‘low’, ‘medium’, or ‘high’ using a composite measure of potential ‘severity’ and ‘likelihood’ of harm. Consensus of independent ranking from three or more panel members was used to determine the final measure of potential harm. Where there was disagreement between individual rankings, a roundtable panel discussion was held to facilitate consensus.

#### 4.2.2. Medication Regimen Complexity

Medication regimen complexity was explored among the intervention group only, as the process of medication reconciliation allowed for comprehensive review of consumed medications. Regimen complexity was characterized by: (i) exploring the types and frequency of changes to the patients’ regimen between timepoints (classified as: new, restarted, dose/frequency increase, dose/frequency decrease, ceased by a medical practitioner (including completion of a treatment course), ceased by the patient (non-adherence), and ‘other’ changes); and (ii) applying the Medication Regimen Complexity Index (MRCI) tool [26].

The overall MRCI score is a summation of complexity derived from three aspects of the regimen: the formulation (A), frequency directions (B), and additional instructions for use (C). Section A was calculated at the regimen level (i.e., patients received a score for different formulations, but each formulation only counted once towards the overall score). Oral tablets and capsules were assigned a base score of one, and other formulations received an increasing score according to the level of complexity required to administer a dose (e.g., liquids scored two points and inhalers scored up to five points depending on device). Section B and C scores were calculated for individual medications (i.e., each medication contributed to the overall score). ‘Once a day’ was assigned a base score of one in Section B, with increasing scores assigned for medicines that were taken more than once a day or at time-specific intervals. Section C scores were calculated based on the number and complexity of additional directions associated with each medication, such as the need to split tablets, vary the dose, or take doses with respect to food. Medicines that were currently withheld under direction of a medical practitioner were included, but no item score for section B or C was assigned (section A scores were included if the formulation was unique). As the MRCI score considers formulation (section A) complexity at the regimen level, medication-level analysis was restricted to section B and C complexity only. For medication-level analysis of complexity (frequency of changes and MRCI score), each medication was scored independently at each time point.

#### 4.2.3. Clinical and Demographic Information

Place of residence was categorized according to the Index of Relative Socioeconomic Disadvantage (IRSD) and the Accessibility/Remoteness Index of Australia (ARIA) [13,14]. Liver disease severity was classified using the Child–Pugh and Model for End-stage Liver Disease (MELD) scores. Comorbidity burden was measured using the Charlson Comorbidity Index [27]. All hospital admissions during the follow-up period were independently reviewed by K.H. and E.P. Encounters were categorized as elective or unplanned, liver-related or non-liver related, medication- or non-medication related, and preventable or non-preventable, as previously reported [12]. Where discrepancies arose, medical records were jointly reviewed, and a discussion was held to facilitate consensus.

### 4.3. Statistical Analysis

Normally distributed data (assessed using the Shapiro–Wilk test) are presented as mean ± standard deviation, or median [interquartile range (Tukey’s Hinges)] if non-normally distributed. Comparisons between groups were made using the independent-samples *t*-test or Mann–Whitney U test (if non-normally distributed). Categorical data are presented as counts and proportions, and analysed using the Pearson’s chi-squared or Fisher’s exact test (if expected cell size ≤ 5). The primary study outcome was the number of discrepancies between patient-reported and clinician-documented ‘current’ medications. A generalized linear model with negative binomial distribution and log link was used to examine factors associated with the incidence rate of discrepancies. Variables that were different between intervention and usual care groups at recruitment, and those associated with total and ‘high-risk’ discrepancies at baseline (*p* ≤ 0.200), were systematically entered into a generalized estimating equations model. Number of discrepancies was offset by number of medications in the final model. The quasi-likelihood under the independence model criterion was used to measure goodness of fit for the final multivariable models. An unstructured correlation matrix with a negative binomial distribution family and log link was used. Incidence rate ratios and 95% confidence intervals are presented. Linear combination of estimators was used to calculate IRRs of discrepancies between covariates (time and randomization). Logistic regression was used to determine predictors of medication-related hospitalization and mortality. Multivariable models were constructed using stepwise conditional backward regression of variables associated (*p* ≤ 0.200) with outcomes on univariate analysis. Child–Pugh score was entered as a continuous variable (possible range 5–15). Odds ratios, adjusted odds ratios, and 95% confidence intervals are reported. Analyses were conducted using IBM SPSS Version 27.0 and Stata Version 15.0.

## 5. Conclusions

The relationship between medication discrepancies and medication-related harm in people with decompensated cirrhosis is complex. Medication reconciliation may improve medication safety by facilitating communication between patients (and their caregivers) and clinicians about ‘current’ therapies, particularly non-CLD medicines, and may help to identify potentially inappropriate medicines and MRPs that could contribute to hospitalization. Pharmacist-led medication reconciliation is effective to reduce discrepancies in people with decompensated cirrhosis and should be considered for implementation in outpatient clinics to improve medication safety.

## Figures and Tables

**Table 1:** Clinical and demographic characteristics of study participants.

Clinical and Demographic Characteristics		Intervention *n* = 57	Usual Care *n* = 57	*p*
Age (mean ± SD)		58.1 ± 10.0	59.5 ± 10.4	0.471 ^1^
Male gender		39 (68.4%)	36 (63.2%)	0.554 ^2^
Liver disease etiology	Alcohol-related liver disease	22 (38.6%)	32 (56.1%)	0.220 ^3^
	Hepatitis C	21 (36.8%)	17 (29.8%)	
	Non-alcoholic fatty liver disease	8 (14.0%)	6 (10.5%)	
	Other	6 (10.5%)	2 (3.5%)	
MELD ^ score (median [IQR])		14.5 [10.5–18.0]	13.0 [10.0–16.0]	0.189 ^4^
Child-Pugh ^ score (median [IQR])		8.0 [7.0–9.0]	8.0 [6.0–9.0]	0.088 ^4^
Ascites at t_0_ (including suppressed by medication)		45 (78.9%)	44 (77.2%)	0.821 ^2^
Encephalopathy at t_0_ (including suppressed by medication)		23 (40.4%)	17 (29.8%)	0.239 ^2^
Variceal bleeding (in the preceding two years)		7 (12.3%)	11 (19.3%)	0.304 ^2^
Hepatocellular carcinoma		4 (7.0%)	11 (19.3%)	0.094 ^3^
Number of medications (median [IQR])	Total	10.0 [7.0–12.0]	8.0 [6.0–9.0]	0.006 ^4^
	CLD	3.0 [2.0–4.0]	2.0 [1.0–3.0]	0.014 ^4^
	Non-CLD	7.0 [4.0–9.0]	6.0 [4.0–7.0]	0.061 ^4^
Medication management *	Self-managed	34 (59.6%)	44 (77.2%)	0.144 ^3^
	Professional caregiver and/or professionally-packed dosage administration aid	9 (15.8%)	4 (7.0%)	
	Partner, family or another caregiver helps	14 (24.6%)	9 (15.8%)	
Charlson Comorbidity Index (median [IQR])		4.0 [4.0–5.0]	4.0 [3.0–9.0]	0.566 ^4^
Highest level of education ^†^	Nil, primary, middle school	26 (53.1%)	18 (32.7%)	0.036 ^2^
	Completed high school and/ or additional education	23 (46.9%)	37 (67.3%)	
Employment status ^‡^	Employed	11 (21.6%)	8 (14.3%)	0.325 ^2^
	Government welfare	37 (72.5%)	45 (80.4%)	0.340 ^2^
	No active income	4 (7.8%)	4 (7.1%)	1.000 ^3^
ARIA	Living in “highly accessible” areas	53 (93.0%)	47 (82.5%)	0.152 ^3^
	Living in “accessible” to “remote” areas	4 (7.0%)	10 (17.5%)	
IRSD	Living in “most disadvantaged” areas	18 (31.6%)	20 (35.1%)	0.691 ^2^
	Living in areas of “low” to “moderate” disadvantage	39 (68.4%)	37 (64.9%)	

Data presented are counts and proportions (%) unless otherwise specified and analysed using the: ^1^ Independent samples *t*-test; ^2^ Pearson’s chi-squared test; ^3^ Fisher’s exact test; ^4^ Mann–Whitney U test. ^ Excludes one intervention patient with no pathology for >6 months due to nonadherence. * Professionally packaged dose administration aids included Webster-pak and multi-dose medication sachet systems. ^†^ Excludes two usual care and eight intervention patients who did not report this information. “Additional education” included a trade qualification, certificate, diploma, or university degree. ^‡^ Excludes one usual care and six intervention patients who did not report this information. Two patients reported concurrent part-time employment and government welfare support and are represented twice. “Employed” includes full-time, part-time, casual, and self-employment. “Government welfare” includes disability support, aged pension, caregiver’s pension, total permanent disability payments, and Newstart allowance. Place of residence was categorized according to the Index of Relative Socioeconomic Disadvantage (IRSD) and the Accessibility/Remoteness Index of Australia (ARIA) [13,14]. Abbreviations: CLD, chronic liver disease; IQR, interquartile range; MELD, model for end–stage liver disease; SD, standard deviation.

**Table 2:** Factors associated with medication discrepancies at baseline.

Variables	Total Discrepancies	*p*	Total ‘High’ Risk Discrepancies	*p*
	IRR (95%CI)		IRR (95%CI)	
Number of medications	1.12 (1.06–1.18)	<0.001	1.10 (1.04–1.17)	0.001
Self-managing medications	0.67 (0.44–1.02)	0.062	0.67 (0.42–1.09)	0.106
Child–Pugh score	1.05 (0.94–1.17)	0.377	1.09 (0.96–1.23)	0.200
Ascites at t_0_	1.41 (0.87–2.28)	0.162	1.70 (0.95–3.06)	0.074
HE at t_0_	1.37 (0.91–2.06)	0.132	1.61 (1.01–2.57)	0.045

**Table 3:** Proportion of discordant medications within intervention and usual care groups at baseline and follow-up.

Medication and Discrepancy Types		Baseline			Follow-Up		
		Usual Care	Intervention	*p*	Usual Care	Intervention	*p*
Total *n* medications ^1^		*n* = 451	*n* = 468		*n* = 310	*n* = 326	
All discrepancies	Name level	45.0%	45.9%	0.777	59.0%	42.6%	< 0.001
	Total	81.8%	77.1%	0.079	87.4%	67.5%	< 0.001
‘High’ risk discrepancies	Name level	15.3%	16.5%	0.632	23.3%	14.7%	0.005
	Total	24.4%	23.3%	0.696	28.6%	18.7%	0.003
CLD medications ^2^		*n* = 118	*n* = 143		*n* = 82	*n* = 96	
CLD discrepancies	Name level	38.1%	34.3%	0.517	52.4%	36.5%	0.032
	Total	71.2%	65.0%	0.290	79.3%	57.3%	0.002
Non-CLD medications ^3^		*n* = 333	*n* = 325		*n* = 228	*n* = 230	
Non-CLD discrepancies	Name level	47.4%	51.1%	0.352	61.4%	45.2%	0.001
	Total	85.6%	82.5%	0.274	90.4%	71.7%	< 0.001

Data presented as count (*n*) of medications within subgroup and proportion (%) that were discrepant. ‘Total’ discrepancies include those at the name and dose/frequency level. Differences between groups was analysed using the Pearson’s chi-squared test. ^1^ Patients taking ≥1 medication at baseline *n* = 114 (intervention *n* = 57; usual care *n* = 57) and follow-up *n* = 78 (intervention *n* = 37; usual care *n* = 41); ^2^ Patients taking ≥ 1 CLD medication at baseline *n* = 105 (intervention *n* = 54; usual care *n* = 51) and follow-up *n* = 73 (intervention *n* = 36; usual care *n* = 37); ^3^ Patients taking ≥ 1 non-CLD medication at baseline *n* = 112 (intervention *n* = 56; usual care *n* = 56) and follow-up *n* = 78 (intervention *n* = 37; usual care *n* = 41).

**Table 4:** Medication Regimen Complexity Index score over time among intervention participants.

MRCI Score	t_0_ (*n* = 57)	t_1_ (*n* = 51)	t_2_ (*n* = 44)	t_3_ (*n* = 39)
Time since t_0_ (weeks)	-	5.7 ± 1.6	14.7 ± 2.1	29.8 ± 5.3
Total MRCI score	25.59 ± 13.49	25.50 ± 13.57	23.42 ± 11.83	24.98 ± 11.70
Section A score	5.12 ± 3.80	5.27 ± 4.53	5.00 ± 4.32	4.77 ± 4.19
Section B score	10.89 ± 5.73	10.95 ± 6.15	9.97 ± 5.53	10.90 ± 5.47
*CLD medicines*	3.38 ± 2.40	3.65 ± 2.57	3.66 ± 2.76	3.62 ± 2.49
*Non-CLD medicines*	7.51 ± 4.98	7.30 ± 5.11	6.31 ± 4.65	7.28 ± 4.63
Section C score	9.58 ± 5.56	9.27 ± 5.08	8.45 ± 4.35	9.21 ± 4.29
*CLD medicines*	2.81 ± 2.33	2.78 ± 2.22	2.95 ± 2.62	2.79 ± 1.98
*Non-CLD medicines*	6.77 ± 4.70	6.49 ± 4.44	5.50 ± 3.50	6.41 ± 3.74

Data presented are mean ± standard deviation. Italicized variables indicate section B and C scores calculated only for CLD and non-CLD medicines.

**Table 5:** Adjusted odds of having ≥ 1 unplanned medication-related hospital admission.

Variable	Adj-OR ^1^ (95%CI)	*p*
Number of ‘high’ risk discrepancies at t_0_	1.25 (0.97–1.63)	0.088
Child–Pugh score	1.35 (1.04–1.75)	0.024
Variceal bleeding	4.85 (1.54–15.28)	0.007
Randomization: intervention	0.79 (0.32–1.99)	0.622

^1^ The final model included 113 cases (*n* = 1 intervention patient missing Child–Pugh score) and was adjusted for total number of ‘high’ risk medication discrepancies at baseline, Child–Pugh score, history of variceal bleeding, and randomization to the intervention group. Abbreviation: adj-OR, adjusted odds ratio.

## Data Availability

The data presented in this study are available on request from the corresponding author. The data are not publicly available as the participants did not consent to its release.

## Supplementary Materials

The following are available online at https://www.mdpi.com/article/10.3390/ph14121207/s1, Table S1: Frequency and type of changes made to intervention patients’ medications throughout the study period; Table S2: Average Medication Regimen Complexity Index Section B and C scores among intervention patients’ medications across all timepoints.

## Appendix A

The clinical significance of discrepancies was classified by the clinician panel using the following assumptions and rules:

1. If the medication was named by the patient but not the clinician, it is assumed that:
  - The patient is taking the medication; and
  - The clinician is not aware that the patient is taking the medication; and
  - Overall risk is assessed on the premise that the patient continues to take the medication without monitoring for three months.

2. If the medication was named by the clinician but not the patient, it is assumed that:
  - The clinician believes the patient is taking the medication; and
  - The patient is not taking the medication; and
  - Overall risk is assessed on the premise that the patient does not take the medication for three months.

3. If the medication is named by both the patient and clinician, but there is a discrepancy between dose/frequency, it is assumed that:
  - The patient is taking the medication; and
  - The clinician believes the patient is taking the medication; and
  - Overall risk is assessed on the premise that the patient takes a ‘standard’ dose (for the patient’s indication) of the medication for three months, as opposed to the dose/s listed by the patient and/or clinician, with concurrent standard-of-care monitoring.

**Table A1:** Risk matrix.

SEVERITY						
		Negligible	Minor	Moderate	Severe	Catastrophic
LIKELIHOOD	Certain	7	11	17	23	25
	Probable	6	10	16	20	24
	Possible	3	9	15	18	22
	Unlikely	2	8	12	14	21
	Rare	1	4	5	13	19

**Risk categorization**

Score 1–5:	Low risk
Score 6–14:	Medium risk
Score 15–22	High risk
Score 23–25:	Very high risk

**Severity definitions**

Negligible:	No harm
Minor:	Minimal harm, managed in general practice/outpatient setting
Moderate:	Temporary harm, including need for additional investigation, minor surgical intervention, or possible hospital admission without life-altering consequences
Severe:	Permanent harm requiring hospital admission with potential for life-altering consequences
Catastrophic:	Permanent harm, including preventable loss of life, intensive care unit admission, or probable life-altering consequences

**Likelihood definitions**

Rare:	The harm would only occur in exceptional circumstances.
Unlikely:	The harm could occur at some time but is not expected (<3 0% of the time).
Possible:	The harm could be expected to occur at some time (30–60% of the time).
Probable:	The harm will likely occur at some time (60–90% of the time).
Certain:	The harm is either occurring or will likely occur in most circumstances (> 90% of the time).

**Table A2:** Rules and examples of medication discrepancy risk classification.

Medication Group	NAME Discrepancy SEVERITY and LIKELIHOOD		Exceptions *(Require Panel Review)*	DOSE/FREQUENCY Discrepancy
	*Patient Named Only*	*Clinician Named Only*		
Diuretics (‘Standard’ dose is spironolactone 100 mg daily/frusemide 40 mg daily)	Severe/Possible (e.g., electrolyte derangement, dehydration)	Moderate/Possible (e.g., admission to hospital with fluid overload)	Not prescribed for ascites/pleural effusion	If standard dose: Minor/Possible If non-standard dose: Panel review
Lactulose	Severe/Unlikely (e.g., electrolyte derangement, dehydration, but less likely than with diuretics because patients less likely to take excessively due to diarrhea)	Severe/Possible (e.g., admission to hospital with encephalopathy)	No history of encephalopathy	All doses: Panel review
Propranolol	Moderate/Possible (e.g., dizziness, hypotension, falls)	Catastrophic/Possible (e.g., variceal bleeding)	No history of varices/grade 1 only	All doses: Panel review
SBP Prophylaxis	Moderate/Possible	Severe/Possible		If standard dose: Minor/Unlikely If non-standard dose: Panel review
Insulins	Severe/Probable (e.g., hypo/hyperglycemia)	Severe/Possible (e.g., Hyperglycemia, uncontrolled diabetes, risk of infection with elevated blood glucose)		All doses: Panel review
Vitamin D (‘Standard’ dose is 25 mcg daily (one tablet))	Minor/Rare	Severe/Rare (e.g., fracture, but rare likelihood of occurrence in three months in people WITHOUT osteoporosis)	Hx of osteoporosis/osteopenia	If standard dose: Negligible/Rare If non-standard dose: Minor/Unlikely
Proton pump inhibitors (‘Standard’ dose is pantoprazole 40 mg daily or equivalent)	Moderate/Unlikely	Minor/Possible if Rx for GORD Other indications: Panel review		If standard dose: Negligible/Rare If non-standard dose: Minor/Possible
Paracetamol (‘Standard’ dose is ≤ 2 g daily)	Minor/Unlikely	Minor/Unlikely	Patient is taking non-standard dose	If standard dose: Negligible/Rare If non-standard dose: Panel review
Inhalers	Minor/Unlikely	Moderate/Possible		If standard dose: Negligible/Rare If non-standard dose: Panel review
Topical (medicated)	Minor/Unlikely	Minor/Unlikely		All PRN doses: Minor/Unlikely All other doses: Panel review

## References

[1] Coletti D.J., Stephanou H., Mazzola N., Conigliaro J., Gottridge J., Kane J.M. (2015). Patterns and predictors of medication discrepancies in primary care. J. Eval. Clin. Pract..

[2] Stewart A.L., Lynch K.J. (2012). Identifying discrepancies in electronic medical records through pharmacist medication reconciliation. J. Am. Pharm. Assoc..

[3] Hayward K.L., Valery P.C., Cottrell W.N., Irvine K.M., Horsfall L.U., Tallis C.J., Chachay V.S., Ruffin B.J., Martin J.H., Powell E.E. (2016). Prevalence of medication discrepancies in patients with cirrhosis: A pilot study. BMC Gastroenterol..

[4] Bedell S.E., Jabbour S., Goldberg R., Glaser H., Gobble S., Young-Xu Y., Graboys T.B., Ravid S. (2000). Discrepancies in the use of medications: Their extent and predictors in an outpatient practice. Arch. Intern. Med..

[5] Franco J.V.A., Terrasa S.A., Kopitowski K.S. (2017). Medication discrepancies and potentially inadequate prescriptions in elderly adults with polypharmacy in ambulatory care. J. Fam. Med. Prim. Care.

[6] Rose O., Jaehde U., Koberlein-Neu J. (2018). Discrepancies between home medication and patient documentation in primary care. Res. Soc. Adm. Pharm..

[7] Perry T.D., Nye A.M., Johnson S.W. (2017). Medication discrepancy rates among Medicaid recipients at hospital discharge. J. Am. Pharm. Assoc..

[8] Coleman E.A., Smith J.D., Raha D., Min S.J. (2005). Posthospital medication discrepancies: Prevalence and contributing factors. Arch. Intern. Med..

[9] Neumiller J.J., Setter S.M., White A.M., Corbett C.F., Weeks D.L., Daratha K.B., Collins J.B., Battles J., Azam I., Reback K., Grady M. (2017). Medication Discrepancies and Potential Adverse Drug Events during Transfer of Care from Hospital to Home.

[10] Hayward K.L., Weersink R.A. (2020). Improving medication-related outcomes in chronic liver disease. Hepatol. Commun..

[11] World Health Organisation (2014). The High 5s Project. Assuring Medication Accuracy at Transitions of Care: Medication Reconciliation.

[12] Hayward K.L., Patel P.J., Valery P.C., Horsfall L.U., Li C.Y., Wright P.L., Tallis C.J., Stuart K.A., Irvine K.M., Cottrell W.N. (2019). Medication-related problems in outpatients with decompensated cirrhosis: Opportunities for harm prevention. Hepatol. Commun..

[13] Australian Bureau of Statistics (2011). Census of Population and Housing: Socio-Economic Indexes for Areas (SEIFA), Australia. http://www.abs.gov.au/AUSSTATS/abs@.nsf/DetailsPage/2033.0.55.0012011?OpenDocument.

[14] Australian Institute of Health and Welfare Rural, Regional and Remote Health: A Guide to Remoteness Classifications. http://www.aihw.gov.au/publication-detail/?id=6442467589.

[15] Lee K.P., Nishimura K., Ngu B., Tieu L., Auerbach A.D. (2014). Predictors of completeness of patients’ self-reported personal medication lists and discrepancies with clinic medication lists. Ann. Pharm..

[16] Weersink R.A., Taxis K., Drenth J.P.H., Houben E., Metselaar H.J., Borgsteede S.D. (2019). Prevalence of drug prescriptions and potential safety in patients with cirrhosis: A retrospective real-world study. Drug Saf..

[17] Nguyen J.K., Fouts M.M., Kotabe S.E., Lo E. (2006). Polypharmacy as a risk factor for adverse drug reactions in geriatric nursing home residents. Am. J. Geriatr. Pharm..

[18] Schoonover H., Corbett C.F., Weeks D.L., Willson M.N., Setter S.M. (2014). Predicting potential postdischarge adverse drug events and 30-day unplanned hospital readmissions from medication regimen complexity. J. Patient Saf..

[19] Bryant B.M., Libby A.M., Metz K.R., Page R.L., Ambardekar A.V., Lindenfeld J., Aquilante C.L. (2016). Evaluating patient-level medication regimen complexity over time in heart transplant recipients. Ann. Pharm..

[20] Metz K.R., Fish D.N., Hosokawa P.W., Hirsch J.D., Libby A.M. (2014). Patient-level medication regimen complexity in patients with hiv. Ann. Pharm..

[21] Libby A.M., Fish D.N., Hosokawa P.W., Linnebur S.A., Metz K.R., Nair K.V., Saseen J.J., Vande Griend J.P., Vu S.P., Hirsch J.D. (2013). Patient-level medication regimen complexity across populations with chronic disease. Clin. Ther..

[22] Cobretti M.R., Page M.R., Linnebur S.A., Deininger K.M., Ambardekar A.V., Lindenfeld J., Aquilante C.L. (2017). Medication regimen complexity in ambulatory older adults with heart failure. Clin. Interv. Ageing.

[23] Thomson M.J., Lok A.S.F., Tapper E.B. (2021). Appropriate and potentially inappropriate medication use in decompensated cirrhosis. Hepatology.

[24] Weersink R.A., Burger D.M., Hayward K.L., Taxis K., Drenth J.P.H., Borgsteede S.D. (2020). Safe use of medication in patients with cirrhosis: Pharmacokinetic and pharmacodynamic considerations. Expert Opin. Drug Metab. Toxicol..

[25] Williams S., Louissaint J., Nikirk S., Bajaj J.S., Tapper E.B. (2021). Deprescribing medications that may increase the risk of hepatic encephalopathy: A qualitative study of patients with cirrhosis and their doctors. United Eur. Gastroenterol. J..

[26] George J., Phun Y.T., Bailey M.J., Kong D.C., Stewart K. (2004). Development and validation of the medication regimen complexity index. Ann. Pharm..

[27] Charlson M.E., Pompei P., Ales K.L., MacKenzie C.R. (1987). A new method of classifying prognostic comorbidity in longitudinal studies: Development and validation. J. Chronic Dis..

