# Supplementary material for: Medication Discrepancies and Regimen Complexity in Decompensated Cirrhosis: Implications for Medication Safety
Source: Pharmaceuticals (Basel). 2021 Nov 23;14(12):1207. doi: 10.3390/ph14121207 (PMC8703811; doi:10.3390/ph14121207)
Supplement: Supplementary file 1 [file pharmaceuticals-14-01207-s001.zip › pharmaceuticals-1458860-supplementary.pdf]

Medication discrepancies and regimen complexity in decompensated cirrhosis: implications for medication safety

Kelly L Hayward, Patricia C Valery, Preya J Patel, Leigh U Horsfall, Penny L Wright, Caroline J Tallis, Katherine A Stuart, Michael David, Katharine M Irvine, Neil Cottrell, Jennifer H Martin, Elizabeth E Powell.

Supplementary Table S1. Frequency and type of changes made to intervention patients' medications throughout the study period.

Medication	Medication Count^	Number of changes (%)	Dose change (%*)	Doctor ceased (%*)	Self-ceased (%*)	New / Restarted (%*)	Other (%*)
All medications	1650	443 (26.8%)	124 (28.0%)	81 (18.3%)	48 (10.8%)	153 (34.5%)	37 (8.4%)
Spironolactone	138	31 (22.6%)	23 (74.2%)	1 (3.2%)	1 (3.2%)	6 (19.4%)	0 (0.0%)
Frusemide	90	25 (27.8%)	15 (60.0%)	3 (12.0%)	1 (4.0%)	6 (24.0%)	0 (0.0%)
Lactulose	80	40 (50.0%)	19 (47.5%)	1 (2.5%)	9 (22.5%)	10 (25.0%)	1 (2.5%)
Propranolol	73	7 (9.6%)	3 (42.9%)	1 (14.3%)	0 (0.0%)	3 (42.9%)	0 (0.0%)
Proton pump inhibitors	113	15 (13.3%)	5 (33.3%)	6 (40.0%)	0 (0.0%)	4 (26.7%)	0 (0.0%)
Inhalers	71	20 (28.2%)	2 (10.0%)	4 (20.0%)	4 (20.0%)	8 (40.0%)	2 (10.0%)
Insulin	39	19 (48.7%)	12 (63.2%)	2 (10.5%)	5 (26.3%)	0 (0.0%)	0 (0.0%)
Prednisolone	10	7 (70.0%)	6 (85.7%)	1 (14.3%)	0 (0.0%)	0 (0.0%)	0 (0.0%)
Analgesics (incl opioids)	133	42 (31.8%)	8 (19.0%)	9 (21.4%)	1 (2.4%)	19 (45.2%)	5 (11.9%)
Benzodiazepines	25	11 (44.0%)	1 (9.1%)	3 (27.3%)	0 (0.0%)	5 (45.5%)	2 (18.2%)
Vitamins / Supplements	356	91 (25.6%)	11 (12.1%)	16 (17.6%)	21 (23.1%)	40 (44.0%)	3 (3.3%)
Cardiovascular	62	10 (16.4%)	2 (20.0%)	3 (30.0%)	1 (10.0%)	3 (30.0%)	1 (10.0%)
Antiemetics	23	9 (39.1%)	1 (11.1%)	1 (11.1%)	0 (0.0%)	5 (55.6%)	2 (22.2%)
Antidepressants / Antipsychotics	78	10 (12.8%)	6 (60.0%)	1 (10.0%)	0 (0.0%)	3 (30.0%)	0 (0.0%)
Topicals	50	18 (36.0%)	2 (11.1%)	1 (5.6%)	0 (0.0%)	5 (27.8%)	10 (55.6%)
Others	309	88 (28.5%)	8 (9.1%)	28 (31.8%)	5 (5.7%)	36 (40.9%)	11 (12.5%)

^ Medications identified at each time point were treated independently. For example, if a patient was taking spironolactone at four timepoints, this medication is represented four times. A total of 1650 medication entries were identified among intervention patients throughout the study period. *Percentage of total medication changes within medication category.

Supplementary Table S2. Average MRCI section B and C scores among intervention patients' medications across all timepoints.

Medication	Medication count^	B	C	Composite B and C
Spironolactone	138	1.08 ± 0.30	1.41 ± 0.77**	2.49 ± 0.75**
Frusemide	90	1.12 ± 0.33	1.26 ± 0.65*	2.38 ± 0.84
Lactulose	80	2.03 ± 1.22**	1.20 ± 0.40*	3.23 ± 1.10**
Propranolol	73	1.93 ± 0.25**	0.55 ± 0.83**	2.48 ± 0.84*
Prednisolone	10	1.00	4.10 ± 1.10**	5.10 ± 1.10**
SBP Prophylaxis	36	1.00	2.08 ± 0.28**	3.08 ± 0.28**
Thyroxine	23	1.00	2.57 ± 0.79**	3.57 ± 0.79**
Insulin	39	1.90 ± 0.79**	1.77 ± 1.09**	3.67 ± 1.63**
Oral hypoglycaemics	47	1.34 ± 0.48	2.06 ± 0.67**	3.40 ± 0.65**
Other medications	1114	1.15 ± 0.56	0.93 ± 0.66	2.08 ± 0.77

Differences between groups were analysed using the Kruskal-Wallis test (pairwise comparisons). Section B scores represent administration frequency complexity. Section C scores represent 'additional instruction' complexity. * $p \leq 0.010$ compared to 'Other medications'; ** $p \leq 0.001$ compared to 'Other medications'.
